# Does paradoxical leadership influence employees’ proactive work behavior? A study based on employees in Chinese state-owned enterprises

**DOI:** 10.3389/fpsyg.2023.1269906

**Published:** 2023-12-18

**Authors:** Qin Qiang, Wu Xiaohong, Song Qianru

**Affiliations:** ^1^School of Journalism and Communication, Renmin University of China, Beijing, China; ^2^Xi’an Technology and Business College, Xi’an, China

**Keywords:** paradoxical leadership, superior-subordinate guanxi, self-efficacy, employees’ proactive work behaviors paradoxical leadership, employees’ proactive work behaviors

## Abstract

Paradoxical leadership has emerged as an increasingly important research topic in the context of Chinese state-owned enterprises, which are currently facing contradictions between maintaining stability and implementing changes, short-term profits and long-term sustainable development, and public nature and marketization. Based on social cognitive theory and social exchange theory, this study employed a questionnaire survey to explore the influence of paradoxical leadership on employees’ proactive work behavior and the mediating role of superior-subordinate guanxi and self-efficacy. The study involved 540 employees working in Chinese state-owned enterprises. We conducted confirmatory factor analyses to test the validity of the measurement model and regression to evaluate the direct effects. Subsequently, we used bootstrapping to confirm mediation and serial mediation effects. The study found that (1) Paradoxical leadership can effectively enhance employees’ proactive work behavior; (2) The superior-subordinate guanxi plays a mediating role between paradoxical leadership and employees’ proactive work behavior, that is, paradoxical leadership enhances employees’ proactive work behavior by improving the superior-subordinate guanxi; (3) Self-efficacy plays a mediating role between paradoxical leadership and employees’ proactive work behaviors, that is, paradoxical leadership promotes employees’ proactive work behavior by enhancing their self-efficacy; (4) The superior-subordinate guanxi and self-efficacy play a chain mediating effect between paradoxical leadership and employees’ proactive work behavior, forming a chain of “Paradoxical leadership—Superior-subordinate Guanxi—Self-efficacy—Employees’ proactive work behaviors.” This study enriches the theoretical research on paradoxical leadership and provides suggestions for state-owned enterprises to enhance employees’ proactive work behavior.

## 1 Introduction

In the increasingly complex and volatile competitive environment, the state-owned enterprises in countries across the globe are confronted with formidable challenges (Ansari, 2019; Mariotti and Marzano, 2020). Especially in China, state-owned enterprises not only face the common contradictions and conflicts of organizations, such as the contradiction between implementing changes and maintaining stability (Farjoun, 2010; Hahn and Knight, 2021), and the conflict between short-term profits and long-term sustainable development (Slawinski and Bansal, 2015), but they also face challenges including public nature and profitability, public value and market value (Zhu et al., 2019; Li et al., 2020), which arises from their inherent nature. These demands, though seemingly contradictory, are interdependent, a phenomenon known as “paradox” (Smith and Lewis, 2011). Contradictions and paradoxes have become the “new normal” in the current uncertain organizational environment (Putnam et al., 2016; Stewart et al., 2019). Therefore, the way leaders of state-owned enterprise effectively navigate these contradictions and tensions in an uncertain environment is crucial for the survival and development of the organization, and has become an urgent issue to resolve (Long and Gao, 2019; Dai et al., 2022). Managers need to treat employees equally while considering individual needs; they need to maintain control while allowing for employee flexibility (Li et al., 2018; Pearce et al., 2019; Zhang and Liu, 2022). Therefore, the ability of managers to handle these contradictory challenges is crucial to effective personnel management. To address these issues, Zhang et al. (2015) integrated Western leadership theory with the philosophy of Yin and Yang in traditional Chinese culture, and innovatively proposed the concept of “Paradoxical Leadership,” developing a corresponding scale, which has gradually attracted widespread attention in academia. They defined it as “leaders adopting seemingly competitive but interconnected behaviors, aiming to simultaneously meet competitive demands in work.”

Leadership style is a discipline that studies the relationship between leaders and employees within an organization, focusing on how different leadership styles have varying effects on employee behavior (Grant et al., 2000; Griffin et al., 2007). Paradoxical leadership, as a specific style, exerts significant influence on employee behavior. As an integral component of leadership styles, paradoxical leadership has garnered significant research attention for its impact on employees, such as the influence of paradoxical leadership on employee adaptability (Zhang et al., 2015), employee creativity (Shao et al., 2019; Yang et al., 2019), employee dual behavior (Kauppila and Tempelaar, 2016), team cognition and innovation (Li et al., 2018), strategic agility (Lewis et al., 2014), promoting work performance improvement (She et al., 2017; Meng et al., 2023), and effectively enhancing organizational innovation capability (Zhu et al., 2020), etc.

However, existing studies have scarcely examined how paradoxical leadership influences employee proactive work behavior, especially in the context of state-owned enterprises. Furthermore, the employees in state-owned enterprises experience an inherent contradiction between being proactive at work and the inclination toward “lying flat,” with the latter becoming increasingly pervasive and unable to reflect positive work behaviors. Proactive behaviors of employees are self-driven actions aimed at solving problems from a long-term perspective. They encompass all constructive behaviors actively taken by individuals with the objective of changing within the organizational state (Frese, 1999; Grant et al., 2000; Griffin et al., 2007; Chen et al., 2019). According to social cognitive theory, it is understood that an individual’s behavior is shaped by both personal and social factors. Social environmental factors influence individual cognitive factors, leading individuals to learn by observing their environment and form individual cognitions (Bandura, 2012). Leaders play dual roles, acting not only as individuals but also as organizational structures that influence employees’ behaviors. Individuals adjust their behaviors by observing others, interpreting these behaviors, and adjusting their own behavior based on these interpretations (Liu et al., 2020). In this context, the superior-subordinate guanxi is pivotal, as employees adjust their behaviors based on their interpretation of leadership behavior (Wu et al., 2020; Cao et al., 2022). Consequently, this paper introduces the superior-subordinate guanxi as a mediating variable in the influence of paradoxical leadership on employees’ proactive work behavior. Meanwhile, social exchange theory also suggests that individuals within an organization will reciprocate when they are treated positively (Eisenberger et al., 2004). Employees’ behavior in the organization is grounded in the principle of reciprocity. In the organization, employees anticipate that their input (such as hard work) will be rewarded (such as recognition and rewards) (Niu et al., 2022; Zhang et al., 2022). Self-efficacy plays a key role in this process, as it affects employees’ confidence in their ability to successfully complete tasks and receive rewards (Guo et al., 2022; Jung et al., 2022). Therefore, this paper introduces self-efficacy as a mediating variable between paradoxical leadership and employees’ proactive work behavior. Paradoxical leadership serves as a pivotal environmental determinant. Through engagements with their superiors, employees discern a balance wherein leaders enforce rigorous work standards yet remain receptive to ambiguity. Such leaders deftly uphold their authoritative stance while simultaneously valuing subordinate feedback. This nuanced leadership dynamic acts as a catalyst, stimulating employees’ proactive work behaviors and fostering a heightened inclination for active participation. How, then, does paradoxical leadership affect employees’ proactive work behavior? What roles do the superior-subordinate guanxi and self-efficacy play in this relationship?

Our research contributes in the following ways: First, we have enriched the outcome variables of paradoxical leadership in the state-owned enterprises. Existing studies have discussed its impact on employee adaptability, employee creativity, employee dual behavior, team cognition and innovation, strategic agility, promoting work performance improvement (Lewis et al., 2014; She et al., 2017; Meng et al., 2023). We have combined the relationship between paradoxical leadership and employees’ proactive work behavior to enrich related research. Second, we have drawn on relevant literature in public administration, psychology by introducing the influence of paradoxical leadership on employees’ proactive work behavior into social cognitive theory and social exchange theory; the style of paradoxical leadership is both a social environment and can bring positive interaction to employees, thus verifying the practical significance of the theory. Third, we will verify the mediating role of self-efficacy and superior-subordinate guanxi in the influence of paradoxical leadership on employees’ proactive work behavior, and the chain mediating effect. In light of existing studies, we noted that employees often hesitate to engage in extra-role proactive behaviors due to concerns of overstepping, embodied in the sentiment “less is more.” Therefore, this paper introduces self-efficacy reflecting employees’ confidence and belief in completing tasks, and uses it as a mediating variable. Based on the premise of studying leadership style and employee behavior in state-owned enterprises with the most Chinese characteristics, the introduction of the important mediating factor of SSG in the research (Kumar and Valeri, 2022), helps to bridge the theoretical research gap between paradoxical leadership and employees’ proactive work behavior. Finally, the research purview was expanded to encompass state-owned enterprises. Hitherto scholarship has yet to scrutinize how paradoxical leadership in state-owned enterprises shapes employee conduct. We incorporated paradoxical leadership into the academic inquiry regarding state-owned enterprises, thereby remedying the lacuna in extant research concerning investigation into state-owned enterprises, thereby enriching the understanding of paradoxical leadership.

## 2 Theoretical background and hypotheses

### 2.1 Paradoxical leadership and proactive work behavior

Operating organizations consistently grapple with an array of contradictions, dilemmas, and challenges (Poole and Van de Ven, 1989). From the 1980s onward, these inherent tensions have been framed as ‘organizational paradoxes’ (Smith and Lewis, 2011), a domain increasingly capturing scholarly attention. Intriguingly, these paradoxes embody elements that, while contradictory, are deeply interwoven. Their dualistic relationships remain enduring, perpetually adapting and reshaping (Shao et al., 2019). On individual analysis, each facet of the paradox seems logical. However, when juxtaposed, they often manifest as illogical, conflicting, or even seemingly nonsensical (Smith and Lewis, 2011). Amidst this intricate milieu, paradoxical leadership (PL) emerges as a promising method to navigate and address organizational conundrums (Probst et al., 2011). A deep dive into existing literature reveals the invaluable role of paradoxical thinking in mediating tensions at both team and individual levels, be it the balancing act between centralization and decentralization, stability vs. aggression, or the tug-of-war between efficiency and quality (Smith and Lewis, 2011; Franken et al., 2020; Zhang et al., 2021; Volk et al., 2022). As Havermans et al. (2015) articulates, paradoxical leadership behaviors possess a dynamic fluidity, facilitating adaptability to diverse work contexts and offering a flexible leadership paradigm. Zhang et al. (2015) delineates paradoxical leadership as a leadership paradigm grounded in embracing cognitive contradictions. It adeptly reconciles the divergent needs of both the individual and the organization, perceives the harmonious coexistence of opposites, and tactically addresses the tensions between organizational and individual objectives to foster collective synergy. The dimensions of paradoxical leadership span across: (1) melding self-centric perspectives with an altruistic outlook; (2) deftly navigating between intimacy and detachment; (3) espousing consistent treatment of subordinates while endorsing individual uniqueness; (4) upholding rigorous work standards yet advocating adaptability; and (5) retaining decision-making authority while fostering autonomy (Lewis et al., 2014; Zhang et al., 2015; Kim, 2021).

In organizations, employees’ proactive work behavior is a positive performance characteristic in realizing organizational value and goals (AlEssa and Durugbo, 2022). Being proactive is a self-control process aimed at goal achievement (Grant and Ashford, 2008), representing a process wherein individuals strive to transform the environment to actualize a better future (Smithikrai, 2022). First proposed by Frese (1999), proactive behaviors, also known as active behaviors, are self-driven approach toward problem-solving from a long-term perspective. It represents a spontaneous and foresighted behavior aimed at changing or improving either the situation or the individual, emphasizing the transformation of work situation characteristics by individuals (Frese et al., 2007; Griffin et al., 2007; Smithikrai, 2022). This study posits that proactive work behavior is a proactive response in the context of leader interaction, social environment, and feedback reception.

According to the social cognitive theory, the behavioral patterns exhibited by leaders and their modeling effect in the organizational context directly influence employees’ cognitive assessments and expectation formation, thereby cultivating a sense of identification and support toward the leaders (Zhang et al., 2021; Volk et al., 2022). Additionally, the attitudes and emotional states held by leaders also have an emotional impact on employees’ job performance. The ramifications of Paradoxical Leadership on Employees’ Proactive Work Behavior predominantly materialize in several distinct facets: First, Inclusivity and Equity: A leader’s adeptness in harmonizing personal ambitions with a genuine regard for others can cultivate a workplace environment that champions fairness and inclusiveness. Drawing from the Social Cognitive Theory, it’s postulated that employees frequently mirror their leaders’ actions (Bandura, 1977). Hence, when leaders prioritize and exhibit empathy and consideration, it can act as a catalyst, encouraging employees to engage proactively in their tasks (Hoch et al., 2018). Second, Balanced Relationships: A leader’s finesse in striking a balance between intimacy and professional detachment with team members can amplify feelings of esteem and worth among employees. This relational equilibrium can galvanize proactive participation in tasks (Jia et al., 2021), a notion echoed by the Social Exchange Theory (Blau, 1964). Third, Uniformity with Flexibility: By endorsing a consistent treatment of subordinates yet championing individual uniqueness, leaders can instill a profound sense of equity and contentment among employees. Such an environment can be a strong impetus for proactive engagement in work (Zhang et al., 2015). Fourth, Clarity with Leeway: Leaders who enforce stringent work norms but also offer ample room for adaptability arm their employees with lucid objectives, coupled with the liberty to realize them. This potent combination can significantly boost proactive engagement in tasks (Choi et al., 2015). Fifth, Control with Autonomy: Retaining a grip over pivotal decisions, while simultaneously advocating employee autonomy, can imbue employees with a sense of empowerment. Such empowerment can, in turn, fortify their proactive involvement in work-related activities (Deci et al., 1989). Contemporary empirical investigations further validate the profound influence of paradoxical leadership on EPWB. It has been observed to nurture an environment ripe with innovation and vigor, thereby amplifying proactive behaviors (Zhang et al., 2015; Li et al., 2018; Yang et al., 2019). In light of these insights, the ensuing hypothesis for this research is posited:

H1: The paradoxical leadership positively affect employees’ proactive work behavior.

### 2.2 The mediating role of superior-subordinate guanxi on the relationship between paradoxical leadership and proactive work behavior

The term “Guanxi” defined and nurtured within the Chinese milieu, embodies a two-way bond between superiors and subordinates within an organization, grounded in mutual interests, emotions, and obligatory duties (Chen and Chen, 2004). This superior-subordinate guanxi, a network of instrumental, emotional, and obligatory connections, influence the behavioral expectations and psychological motivations of both superiors and subordinates during interaction (Su et al., 2022; Zhong et al., 2022). In contrast, Western research designates this superior-subordinate dynamic as Leader-Member Exchange (Zhang et al., 2016). However, they have different theoretical connotations. First, the domains of social exchange differ. In Chinese superior-subordinate guanxi, the boundary between public and private tends to blur, extending beyond the work, whereas Leader-Member Exchange confines itself within the professional ambit. Second, the foundation of guanxi is different: superior-subordinate guanxi leans heavily on personal feelings and special connections, often utilizing ties like kinship, relies on special ties and connections, commercial associations, and geographical connections for its establishment (Law et al., 2000; Zhang et al., 2021). Compared to talent and contribution, feelings and loyalty are the criteria that Chinese managers pay more attention to when choosing in-group employees. In contrast, Leader-Member Exchange is predicated upon job performance and resource rewards, prioritizing talent and ability over personal guanxi (Yang et al., 2019). Third, the reciprocity rules in these contexts diverge. The Chinese interaction model emphasizes preserving face and harmony, potentially endorsing favoritism, while Leader-Member Exchange focuses on task and performance, promoting equality and fairness (Ma et al., 2023). Therefore, given the cultural background, employee traits, and organizational environments, indigenous constructs such as superior-subordinate guanxi may offer more applicable management insights and decision-making recommendations for the Chinese context compared to mature Western theories like Leader-Member Exchange.

In daily work, paradoxical leaders who strike a balance between fair treatment and recognition of individua needs (He and Yun, 2022), who listen to employees’ feelings and opinions (Wu et al., 2020), who delegate decision-making for minor issues while retaining control over significant decisions (Le et al., 2020), and who demand consistency yet allow exceptions (Zhao et al., 2023), are likely to win their employees’ approval and trust. This, in turn, facilitates the establishment of robust superior-subordinate guanxi.

The perception of ‘risk’ associated with proactive work behavior is often influenced by the leader’s characteristics and actions (Zhang et al., 2015; Contreras et al., 2020). Employees who have built good superior-subordinate guanxi with leaders enjoy more psychological and cognitive resources, leading them to perceive proactive behaviors as safer (Xue et al., 2020). They also engage in more interactions with leaders, which can foster mutual trust and understanding (Law et al., 2000). Furthermore, strong emotional bonds with leaders encourage employees to assist in problem-solving, stimulating employee’s proactive work behavior (Xiao et al., 2021). In conclusion, the quality of the relationship between leaders and employees can significantly influence the employees’ willingness to take risks and engage in employee’s proactive work behavior.

Social cognitive theory suggests that in terms of observation and learning, managers are also the learning objects of employees within the organization. Employees observe and imitate managers, which influences their personal cognition and thus their behavior (Bandura, 2002; Thomas and Gupta, 2021). Social exchange theory also posits that when employees receive positive feedback from superiors (Cropanzano and Mitchell, 2005; Cortez and Johnston, 2020), they will also provide positive action feedback, leading to proactive work behavior. Paradoxical leadership can handle SSG well and build a harmonious organizational atmosphere (Wang et al., 2019).

Employees’ behavior is influenced by both individual characteristics and leadership behavior (Zhang, 2012; de Jong et al., 2021). Employees who recognize the paradoxical leader’s respect, trust, and support for their self-growth and self-worth may be more inclined to forge positive interpersonal relationships. Harmonious Superior-subordinate guanxi can effectively reduce various concerns about employees’ proactive work behavior, encouraging a more active and initiative-taking approach. Therefore, the following hypothesis is proposed in this study:

H2: Superior-subordinate guanxi mediates the relationship between paradoxical leadership and employees’ proactive work behavior.

### 2.3 The mediating role of self-efficiency on the relationship between paradoxical leadership and proactive work behavior

Self-efficacy denotes an individual’s confidence in their capability to successfully carry out tasks and meet required objectives (Bandura, 1977; Tierney and Farmer, 2002; Trong Tuan, 2017; Boruszak-Kiziukiewicz and Kmita, 2020). It bifurcates into two categories: Firstly, specific self-efficacy, which is an individual’s beliefs about accomplishing a specific let task or work behavior (Bandura, 1977; Ren et al., 2020); Secondly, generalized self-efficacy, a stable individual trait, reflects an individual’s confidence in their ability to meet work requirements in different task situations (Gist, 1987; Morelli et al., 2020). In this research, our focus is on the function of SE in the relationship between leadership style and employees’ proactive work behavior. Given that the organizational context is undefined, we interpret employee self-efficacy as general self-efficacy.

According to social cognitive theory, self-efficacy is a form of personal cognition that affects employee behavior and is influenced by the manner and attributes of managers (Shao et al., 2019; Jung et al., 2022). An individual’s inclination toward proactive work behaviors is contingent upon their faith in the organization and their personal abilities (Hsieh et al., 2016). It is believed that employees’ proactive work behavior, once initiated, are capable of being accomplished by the individual and that the organization provides the appropriate platform to do so (Jia et al., 2021). Paradoxical leadership, with its comprehensive understanding of each employee’s strengths and weaknesses, and the reassurance in its words and actions, effectively mitigates overthinking in employees regarding proactive work behaviors (Chen et al., 2021). This approach enhances employees’ sense of efficacy, and give employees the confidence to accomplish their work, and fosters employees’ proactive work behavior. Accordingly, this study posits the following hypotheses:

H3: Self-efficacy mediates the relationship between paradoxical leadership and employees’ proactive work behavior.

### 2.4 The chain mediating role

Employee’s proactive work behavior is not immediate, it is based on the degree of certainty of their own ability to complete the task and result orientation, essentially, judgment before action (Fay et al., 2023). High-quality superior-subordinate guanxi can make employees feel unsuspecting in the organization, without always worrying about whether their superiors are dissatisfied with their work, and apprehension over peers’ opinions (Hayat Bhatti et al., 2022). This alleviation of concern enables employees to think ahead, focusing on their tasks rather than their perceived probability of success. In alignment with social cognitive theory, high-quality superior-subordinate guanxi, will make employees more trusting of the organization. A healthy organizational climate promotes open communication, facilitating employee understanding of tasks (Han et al., 2019), thereby instilling certainty and confidence in completing the work, which subsequently impact employees’ proactive work behavior. Based on the above discussion proposed as follows:

H4: Self-efficacy mediates between superior-subordinate guanxi and employees’ proactive work behavior.

According to social cognitive theory, employees’ cognition and behavior are affected by their characteristics and interactions with leaders. The most direct product of this process is the superior-subordinate guanxi (Zhao and Zhou, 2021). Paradoxical leadership, which treats subordinates equally yet uniquely, underlines a benign, harmonious superior-subordinate guanxi (Jung et al., 2022). High-quality superior-subordinate guanxi cultivates employees’ trust in the organization, promoting an open communication environment (Ma, 2022), thereby impacting employees’ proactive work behavior. The mediation of self-efficacy between superior-subordinate guanxi and employee constructs suggests that proactive work behavior is contingent on employees’ confidence in their ability to complete tasks and results-oriented focus (Kang and Lee, 2021). Hence, the propensity of employees toward proactive work behavior is intimately tied to their relationships with organizational leaders and colleagues (Virgiawan et al., 2021). Leaders who build better relationships with their employees can inspire employees toward collective efforts and good intra-organizational relations, thus enhancing self-efficacy for employees’ proactive work behavior (Sarwar et al., 2020). Therefore, superior-subordinate guanxi of high quality can completely bring stronger self-efficacy to employees in their proactive work behavior and can promote proactive work behavior (Choi et al., 2021). Given these observations, we postulate a chain mediation effect of superior-subordinate guanxi and self-efficacy between paradoxical leadership and employees’ proactive work behavior, i.e., “paradoxical leadership–superior-subordinate guanxi–sense of self-efficacy–employees’ provocative work behavior.” Some scholars have suggested that there is not only a single link of mediating variables between the independent variable and the dependent variable, but also a chain of mediators with sequential order formed by the combination of different variables exerting mediating effects, which is called chained mediating effect (Wang et al., 2021). Thus, this study proposes the following hypotheses:

H5: Superior-subordinate guanxi and self-efficacy play a chain mediating role between paradoxical leadership and employees’ proactive work behavior.

**Figure 1:**
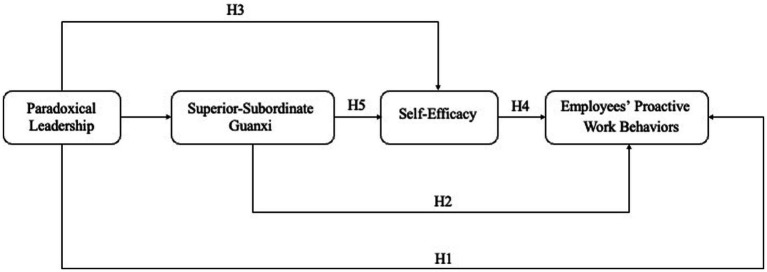
Theoretical model.

This paper takes social exchange theory and social cognitive theory as its basic theory. It provides a comprehensive overview of research on employees’ proactive work behavior, encompassing both role-centric and extra-role work behaviors. Paradoxical leadership behavior will, in essence, provide a suitable “environment” for employees’ proactive work behavior, by giving employees a certain degree of support to ensure the relative freedom of employees’ work, which can effectively balance the competing needs of employees and the organization. As employees’ trust in leadership and the organization heightens, their self-confidence and performance, whether within their role or ‘extra’, improve, contributing positively to the organization. Therefore, by approaching from a dual perspective of social relations and individual psychological mechanisms, we construct a theoretical model as depicted in Figure 1.

## 3 Materials and methods

### 3.1 Participants

This study used a cluster random sampling method. We initially reached out to a contact at the State Assets Supervision and Administration Commission (SASAC). Leveraging this connection, we engaged with 12 state-owned enterprises in Beijing to distributed electronic questionnaires. The survey, conducted in June 2022, saw participation from 773 workers. Questionnaires that were obviously not in accordance with the normal time, missed, and incorrectly filled out were discarded. Ultimately, 540 valid questionnaires were obtained, with an effective recovery rate of 69.86%.

### 3.2 Measures

#### 3.2.1 Paradoxical leadership

We utilized the extensively validated paradoxical leadership scale developed by Zhang et al. (2015), which has proven useful in recent research. The scale consists of five dimensions and contains 22 question items, such as “My supervisor treats all subordinates in a fair manner consistently, while also regarding them as unique individuals,” “My supervisor positions all subordinates on an equal standing, yet also takes into account their distinct attributes and individualities.” Its Cronbach’s α of all five dimensions of paradoxical leadership is greater than 0.8, and the Cronbach’s α of the total scale is >0.9, which can prove that the scale’s reliability.

#### 3.2.2 Employees’ proactive work behavior

This was gaged using the scale developed by Parker and Collins (2010), which encapsulates both role and out-of-role and extra-role proactive behaviors, across four dimensions and 13 question items, such as “At work, I communicate my perspectives to colleagues even when my views diverge from others and encounter some opposition,” “I communicate with others about issues or work assignments that personally impact me in the workplace, and encourage their participation in these matters.” Employees’ Proactive Work Behaviors Scale Cronbach’s α > 0.9, indicating that the Employees’ Proactive Work Behaviors Scale (Formal Version) reliability is excellent.

#### 3.2.3 Superior-subordinate guanxi

To measure this construct, employees were asked to rate their likelihood of exhibiting the behaviors/activities proposed by four items developed by Law et al. (2000). It contains 4 items, such as “My supervisor and I genuinely care about each other like good friends.” “My supervisor and I will share each other’s difficulties and stresses as much as possible.” The scale’s Cronbach’s α is 0.940.

#### 3.2.4 Self-efficacy

Based on the SE theory (Bandura, 1977; Chesney et al., 2006)., this measure focuses on the changes in individuals’ confidence in their ability to cope effectively via10-item unidimensional scale, such as “I can always fix things if I try my best,” “Even if others oppose me, I still have a way to get what I want.” The scale’s Cronbach’s α > 0.9, proving that the self-efficacy scale has good reliability.

In addition, previous studies have indicated that young males with higher education will perform better proactive work behavior (AlEssa and Durugbo, 2022). Also, workers with greater working experiences will perform less proactive behaviors (Smith and Lewis, 2011). Consequently, we included gender, age, education and working experiences as control variables.

Initially, we designed a preliminary questionnaire by adopting established scales based on the literature review and theoretical analysis. Employees self-assessed their leaders’ paradoxical leadership style and their hierarchical relationships. Self-efficacy and EPWB were also self-reported by employees. Following this, we requested peers in the workshop to independently complete the questionnaire and provide their feedback. The questionnaire was then refined by combining the opinions and suggestions. Due to the COVID-19 epidemic, the online questionnaire was conducted using a snowball sampling method. Questionnaires were distributed to university classmates, friends, etc., who were employed in state-owned enterprises. Statistical analysis was carried out on the returned sample data and inappropriate statistical requirement questions in the pre-questionnaire were revised to finalize the official questionnaire.

### 3.3 Analytical strategy

In this study, SPSS 25.0 and Mplus 7.4 were used for data analysis. SPSS was mainly used for data sorting, descriptive statistical analysis, etc. Mplus is mainly used for model inspection, which is used by prior researches (Pan et al., 2022; Wang, 2022). Mplus was chosen for analyzing serial mediation effects due to its specialized expertise in Structural Equation Modeling and its ability to address nuanced methodological considerations inherent in such analyses. It is well-known for its proficiency in handling latent variables and intricate pathways, providing a versatile framework for complex serial mediation modeling. Participants who lacked descriptive data or had many data points were treated by list deletion when running the analysis.

## 4 Results

### 4.1 Descriptive statistics

Tables 1, 2 lists the major variables. Five hundred and forty valid questionnaires were obtained. Among them, 301 (55.7%)were male and 239 (44.3%)were female. Two hundred and fifteen respondents (39.8%) were aged between 25 and 35, while 195 (36.1%) were between 35 and 45. The working experience between 1 and 5 were 223 (41.3%) and 5–10 were 170 (31.5%). Most of them are undergraduate (68.5%). The balanced distribution of gender, education, work experience, among other factors, effectively ensure the sample’s representativeness. As shown in Table 3, there is a significant positive correlation between the five dimensions of paradoxical leadership and SSG, SE, and employees’ provocative work behaviors respectively, at the 0.01 significance level; Similarly, SSG is significantly positively correlated with both SE (*r* = 0.273, *p* < 0.01), and employees’ provocative work behaviors (*r* = 0.271, *p* < 0.01). Furthermore, there exists a significantly positive correlation between SE and employees’ proactive work behavior (*r* = 0.332, *p* < 0.01).

**Table 1:** Descriptive statistics.

Variable	Item	Num	Percentage (%)
Gender	Male	301	55.7
	Female	239	44.3
Age	<25	43	8.0
	25 ~ 35	215	39.8
	35 ~ 45	195	36.1
	>45	87	16.1
Education	College	75	13.9
	Undergraduate	370	68.5
	Master degree	95	17.6
Working experience	<1	66	12.2
	1 ~ 5	223	41.3
	5 ~ 10	170	31.5
	>10	81	15.0

*N* = 540.

**Table 2:** Results of correlation coefficients.

Variable	Mean	SD	1	2	3	4	5	6	7	8	9	10	11	12
UI	4.444	1.337	1											
SO	4.057	1.338	0.493**	1										
CA	4.508	1.391	0.563**	0.413**	1									
RF	4.497	1.398	0.332**	0.284**	0.364**	1								
DC	4.345	1.155	0.313**	0.221**	0.329**	0.619**	1							
SSG	4.788	1.161	0.481**	0.225**	0.428**	0.137**	0.117**	1						
SE	4.974	0.931	0.222**	0.169**	0.233**	0.241**	0.182**	0.273**	1					
EPWB	5.109	0.732	0.238**	0.234**	0.235**	0.304**	0.214**	0.271**	0.332**	1				
Gender	1.443	0.497	−0.086*	−0.040	−0.087*	−0.038	−0.018	−0.170**	−0.084	−0.146**	1			
Age	2.604	0.850	−0.094*	−0.007	−0.053	−0.039	−0.020	−0.056	−0.032	0.007	0.034	1		
Education	2.037	0.560	−0.031	−0.020	0.003	−0.064	−0.052	−0.049	−0.051	−0.014	0.001	−0.094*	1	
Working experience	2.493	0.892	0.0259	−0.023	−0.019	−0.019	−0.046	0.016	−0.061	−0.068	−0.028	−0.038	−0.033	1

*N* = 540; **p < 0.01, *p < 0.05; 1 is male, 2 is female. UI: treating subordinates uniformly, while allowing individualization; SO: combining self-centeredness with other-centeredness; CA: maintaining decision control, while allowing autonomy; RF: enforcing work requirements, while allowing flexibility; DC: maintaining both distance and closeness; SSG: supervisor-subordinate guanxi; SE: self-efficacy; EPWB: employees’ proactive work behavior.

**Table 3:** Comparison of competing CFA model results.

Model	χ^2^/df	CFI	TLI	SRMR	RMSEA (90%CI)
UI, SO, CA, RF, DC, SSG, SE, EPWB	2.387	0.919	0.913	0.070	0.051
UI, SO, CA, RF, DC, SSG + SE, EPWB	4.464	0.869	0.848	0.097	0.083
UI, SO, CA, RF, DC, SSG + EPWB, SE	4.753	0.859	0.854	0.085	0.091
UI, SO, CA, RF, DC, SE + EPWB, SSG	4.853	0.830	0.822	0.084	0.088
UI, SO, CA, RF, DC, SSG + SE + EPWB	5.014	0.805	0.775	0.139	0.124
UI + SO+CA + RF + DC, SSG, SE, EPWB	3.385	0.885	0.875	0.075	0.067
UI + SO+CA + RF + DC + SSG, SE, EPWB	4.953	0.856	0.847	0.093	0.104
UI + SO+CA + RF + DC + SE, SSG, EWB	4.951	0.866	0.857	0.089	0.100
UI + SO+CA + RF + DC, SSG + SE, EPWB	4.784	0.846	0.843	0.084	0.094
UI + SO+CA + RF + DC + EPWB, SSG, SE	7.305	0.721	0.705	0.142	0.137
UI + SO+CA + RF + DC, SSG + EPWB, SE	5.493	0.832	0.819	0.869	0.123
UI + SO+CA + RF + DC, SSG, SE + EPWB	5.743	0.793	0.783	0.105	0.121
UI + SO+CA + RF + DC + SSG + SE, EPWB	6.385	0.758	0.748	0.995	0.125
UI + SO+CA + RF + DC, SSG + SE + EWB	5.596	0.748	0.743	0.108	0.121
UI + SO+CA + RF + DC + SSG, SE + EPWB	6.642	0.726	0.703	0.109	0.131
UI + SO+CA + RF + DC + SE, SSG + EPWB	6.578	0.734	0.722	0.104	0.126
UI + SO+CA + RF + DC + EWB, SSG + SE	7.742	0.692	0.651	0.143	0.148
UI + SO+CA + RF + DC + SSG + EPWB, SE	7.761	0.689	0.632	0.155	0.158
UI + SO+CA + RF + DC + SE + EPWB, SSG	7.859	0.668	0.632	0.167	0.174
UI + SO+CA + RF + DC + SSG + SE + EPWB	8.748	0.622	0.601	0.179	0.186

N =540. UI: treating subordinates uniformly, while allowing individualization; SO: combining self-centeredness with other-centeredness; CA: maintaining decision control, while allowing autonomy; RF: enforcing work requirements, while allowing flexibility; DC: maintaining both distance and closeness; SSG: supervisor-subordinate guanxi; SE: self-efficacy; EPWB: employees’ proactive work behavior.

### 4.2 Model inspection

The model was fitted with Mplus. The results of the confirmatory factor analysis indicated that the fit indices of the eight-factor measurement model were clearly superior compared to alternative models, thus substantiating the discriminant validity of the construct measurements employed in this study (see Table 3).

### 4.3 Hypothesis testing

#### 4.3.1 Assessment of the direct impact of paradoxical leadership on employees’ proactive work behavior

Table 4 provided a comprehensive analysis of the direct effects of paradoxical leadership on employees’ proactive work behavior. Initially, Model 1 examined the influence of gender, age, etc., on proactive work behavior, revealing a significant gender-based difference (β = −0.218, *p* < 0.01); males exhibited more proactive behaviors than females, aligning with previous variance analysis results. Models 2 through 6 assessed the impact of each dimension of paradoxical leadership on proactive work behavior. Findings indicated a significant positive correlation across all five dimensions with employees’ provocative work behavior at the 0.001 significance level, supporting Hypothesis H1. Additionally, this paper also used the tolerance, variance inflation factor (VIF) to test the problem of multicollinearity between all the dimensions of paradoxical leadership and superior-subordinate guanxi. The results, which displayed VIF <10, tolerance >0.1, confirmed that there was no significant co-collinearity problem. Therefore, in Chinese state-owned enterprises, paradoxical leadership can effectively influence employees’ proactive work behavior. This also explains why leadership training programs are frequently organized in Chinese state-owned enterprises, with particular emphasis on aspects of Chinese culture such as heaven and earth, yin and yang, movement and stillness, Dao and technique, firmness and flexibility, and so forth.

**Table 4:** Results of paradoxical leadership and employees’ proactive work behavior.

Variable	Employee’s proactive work behavior					
	Model 1	Model 2	Model 3	Model 4	Model 5	Model 6
Gender	−0.218**	−0.190**	−0.205**	−0.190**	−0.202**	−0.213**
Age	−0.007	−0.026	0.009	−0.017	0.018	0.012
Education	−0.020	−0.008	−0.014	−0.020	0.007	−0.005
Working experiences	−0.059	−0.063	−0.055	−0.055	−0.054	−0.051
UI		0.127***				
SO			0.124***			
CA				0.118***		
RF					0.156***	
DC						0.123***
R2	0.027	0.097	0.078	0.076	0.115	0.070
ΔR2	0.020	0.071***	0.070***	0.068***	0.107***	0.061***
F	3.690	9.203***	9.063***	8.822***	13.893***	8.031***

*N* = 540; ***p < 0.001, *p < 0.05. UI: treating subordinates uniformly, while allowing individualization; SO: combining self-centeredness with other-centeredness; CA: maintaining decision control, while allowing autonomy; RF: enforcing work requirements, while allowing flexibility; DC: maintaining both distance and closeness; SSG: supervisor-subordinate guanxi; SE: self-efficacy; EPWB: employees’ proactive work behavior.

#### 4.3.2 The mediating role and chain mediating role

The result equation model of this paper was developed using Mplus 7.4 to test the mediating effect of SSG on all dimensions of paradoxical leadership and the employee provocative work behaviors, as well as the mediating effect of SE on the five dimensions of paradoxical leadership and employee provocative work behaviors. Furthermore, the study investigated the mediating effect of SE on the link between SSG and employees’ provocative work behaviors, and also explored the chain mediating effect of SSG and SE on the relationship between the 5 dimensions of paradoxical leadership and employees’ provocative work behavior. As evidenced by Table 5, all the fitting indicators of all models fit well, as indicated by their fitting metrics aligning with the predetermined decision values, which sets the stage for subsequent detailed analysis.

**Table 5:** The fit of mediating role and chain mediating role of SSG and SE.

Model	χ^2^/df	CFI	TLI	SRMR	RMSEA (90%CI)
Model 1	2.770	0.919	0.912	0.087	0.057
Model 2	2.568	0.911	0.904	0.075	0.054
Model 3	3.508	0.911	0.901	0.093	0.068
Model 4	2.411	0.915	0.909	0.077	0.051
Recommendations	<5	>0.9	>0.9	<0.1	<0.1
Yes or no	Yes	Yes	Yes	Yes	Yes

Model 1 represented the mediating effects of SSG between all dimensions of paradoxical leadership behavior (PLB) and EPWB; Model 2 denoted the mediating effects of SE between all dimensions of PLB and EPWB; Model 3 represented the mediating effects of SE between SSG and EPWB; and Model 4 represents the chain mediating effects of SSG and SE between all dimensions of PLB and EPWB.

As illustrated in Table 6, Model 1showed a moderated mediating effect value of 0.087, with a confidence interval of [0.0387, 0.1420] that excluded 0. This implied a significant mediating effect, indicating that the supervisor-subordinate guanxi played a mediating role between the paradoxical leadership and the employees’ proactive work behavior, and the hypothesis H2 was verified. In Chinese bureaucratic organizations, the majority of employees in state-owned enterprises tend to follow the directives of their superiors and are significantly influenced by them. If superiors adopt a paradoxical leadership style, it creates a flexible superior-subordinate guanxi, where employees not only comply with the leadership’s directives but are also willing to do so.

**Table 6:** Results of mediating role and chain mediating role.

Model	Effect	Variables	Confidence interval	Effect value	Effect size
Model 1	Direct effect	PLB → EPWB	[0.0023, 0.0283]	0.144	62.338%
	Mediating effect	PLB → SSG → EPWB	[0.0387, 0.1420]	0.087	37.662%
	Total effect		[0.0815, 0.1717]	0.231	100.00%
Model 2	Direct effect	PLB → EPWB	[0.0490, 0.1375]	0.170	73.593%
	Mediating effect	PLB → SE → EPWB	[0.0292, 0.0977]	0.061	26.407%
	Total effect		[0.0815, 0.1717]	0.231	100.00%
Model 3	Direct effect	SSG → SE	[0.0636, 0.1675]	0.183	71.765%
	Mediating effect	SSG → SE → EPWB	[0.0385, 0.1097]	0.072	28.235%
	Total effect		[0.1089, 0.2130]	0.255	100.00%
Model 4	Direct effect	PLB → EPWB	[0.0129, 0.1109]	0.112	48.485%
	Mediating effect	PLB → SSG → EPWB	[0.0134, 0.1143]	0.062	26.840%
		PLB → SE → EPWB	[0.0034, 0.0641]	0.031	13.420%
	Chain mediating effect	PLB → SSG → SE → EPWB	[0.0110, 0.0430]	0.026	11.255%
	Total mediating effect		[0.0655, 0.1738]	0.118	51.082%
	Total effect		[0.0815, 0.1717]	0.231	100.00%

*N* = 540; UI: treating subordinates uniformly, while allowing individualization; SO: combining self-centeredness with other-centeredness; CA: maintaining decision control, while allowing autonomy; RF: enforcing work requirements, while allowing flexibility; DC: maintaining both distance and closeness; SSG: supervisor-subordinate guanxi; SE: self-efficacy; EPWB: employees’ proactive work behavior.

Model 2 displayed a moderated mediating effect value of 0.061 with the confidence interval of [0.0292, 0.0977], did not contain 0, suggesting a significant mediating effect. This indicated that self-efficacy played a mediating role between paradoxical leadership and employees’ provocative work behaviors, thus confirming hypothesis H3. Due to its emphasis on affording employees ample respect, autonomy, and encouragement, paradoxical leadership enables employees to develop a sense of competence in carrying out their tasks effectively. This enhances their self-confidence and cultivates a sense of self-efficacy, subsequently leading to an elevation in employees’ provocative work behavior.

In Model 3, a moderated mediating effect value of 0.072 was observed, with the confidence interval of [0.0385, 0.1097] that excluded 0, which signified a substantial mediating effect. This indicated that SE played a mediating role between supervisor- subordinate guanxi and employees’ provocative work behaviors, thus verifying Hypothesis H4. Model 4 revealed a regulated total mediating effect value of 0.118, the confidence interval is [0.0655, 0.1738] that excluded 0, signifying a significant mediating effect. This indicated that both supervisor- subordinate guanxi and self-efficacy played chain mediating role between paradoxical leadership and employees’ provocative work behavior, thereby supporting hypothesis H5. The establishment of positive superior-subordinate guanxi is often a focal point of attention within state-owned enterprises. A harmonious superior-subordinate guanxi enables employees to perceive their own significance. Leaders, at appropriate junctures, delegate suitable tasks, not only enhancing employees’ sense of well-being and emotional state, but also boosting work efficiency. This, in turn, fosters a heightened sense of self-efficacy among employees. This elucidates why there is presently a heightened emphasis on cultivating positive superior-subordinate guanxi within state-owned enterprises, and why consultations with subordinate employees’ opinions are sought in leadership promotions.

## 5 Discussion

### 5.1 Summary

Synthesizing the above studies, this paper draws the following main research conclusions:

First, the five dimensions of paradoxical leadership have a significant positive impact on employees’ proactive work behavior.

While prior research has primarily focused on the influence of single-type leadership styles, such as people-oriented or abusive ones (Zhu et al., 2020), on employees’ proactive work behaviors, fewer studies have explored the impact of diversified leadership styles, such as paradoxical leadership. This study examines the positive influence of the five significant features of paradoxical leadership style on employees’ active work behavior.

(1) Every employee desires to be recognized and given attention (Guo et al., 2020). Paradoxical leadership can solidify its core influence, shift the focus as needed, while acknowledging the employee’s desire for recognition, and willingly share leadership roles and functions with the team (Kim, 2021). This combination of personal charm and sharing of roles cultivates within employees a strong sense of ownership (Zhang et al., 2015), thereby enhancing their engagement with the organization and reinforcing their commitment to its growth. Consequently, employees are more likely to display proactive work behavior (Le et al., 2020).
(2) Regarding the paradoxical leadership approach of treating subordinates with both equality and individuality, employees can tangibly perceive this unique blend of fairness and respect emanating from leadership and the organization (Zhang et al., 2022). Previous research has shown that leadership’s sense of justice and organizational fairness influence employees’ active work behaviors (Zhao et al., 2020). Concurrently, assigning tasks based on employees’ personality traits, such as action style, personal characteristics, and areas of expertise, while deemphasizing hierarchical differences in status, may enhance trust and promote mutual understanding, leading to an equitable and respectful relationship with staff. This, in turn, encourages employees to prioritize the organization’s interests and address issues for their leaders and colleagues (Shaw et al., 2020).
(3) Ensuring centralized control and autonomy in decision-making, paradoxical leaders keep significant decision-making matters in their own hands to maintain overall organizational control (Zhu et al., 2021). Simultaneously, they delegate authority, allowing employees to make decisions on certain minor or less important tasks. Such delegation implies trust and respect, fostering employees’ confidence and promoting their proactive work behavior (Debebe et al., 2016). Paradoxical leaders offer their employees a certain level of autonomy. This leads to positive thoughts and proactive actions in order to use their power effectively and complete tasks (Casas Klett and Arnulf, 2020). Conversely, if leaders exert monopolistic control, employees become mere followers of orders. They are limited to doing exactly as they are told, which ultimately causes a decline in organizational vitality and hinders employee initiative. And for leaders who prefer to monopolize power, the initiative demonstrated by employees could potentially upset their notion of authority (Rescalvo-Martin et al., 2021). In the face of such a risk, employees might choose to refrain from taking the initiative in order to avoid unnecessary conflicts. This reluctance to take action could stifle creativity and productivity, eventually leading the organization into stagnation (Feng et al., 2020).
(4) Striking a balance between adhering to established plans and maintaining necessary flexibility, paradoxical leadership emphasizes setting clear goals and objectives while also taking into account factors such as task difficulty, time allocation, and individual capabilities (Sparr et al., 2022). This approach allows employees some degree of autonomy and flexibility when tackling their tasks within a relatively supportive environment (Zada et al., 2022). Such an environment can improve employee performance by providing affirmation and encouragement. However, setting excessively high standards or relinquishing control can be counterproductive. The former could lead to burnout due to excessive pressure, while the latter could lead to complacency and a disregard for work quality and progress (Wang et al., 2022). Paradoxical leadership can mitigate negative outcomes by providing employees with flexibility to handle their tasks. Furthermore, as employees gain more hands-on experience and decision-making opportunities, their understanding of leadership deepens, fostering empathy toward leaders’ daily challenges. Employees are more likely to engage in proactive communication and share their insights with leadership, leading to enhanced mutual trust and a stronger relationship. This improved communication and relationship can motivate employees to initiate work behaviors, whether driven by leadership direction or the principle of reciprocity.
(5) In terms of maintaining relational closeness and distance, paradoxical leaders are able to establish clear hierarchical relationships with their employees based on the organizational hierarchy (Xue et al., 2020). However, they are not lacking in emotional closeness toward their employees, and they communicate their kindness. “Distance” serves as the basis for carrying out work tasks in compliance with organizational directives, ensuring the gravity of the work (Qu et al., 2022). Conversely, a “sense of closeness” can foster a harmonious organizational atmosphere. This duality promotes mutual understanding, facilitates exchange of ideas, and increases the likelihood of identifying common ground (Jung et al., 2022). As a result, employees’ trust in the leadership strengthens, leading to a chain reaction where employees take initiative to improve the organization through their own actions, including maintaining a positive work environment and promoting active work behavior (Nevicka et al., 2018).

In light of these observations, the social exchange theory can explain the influence of paradoxical leadership on employees’ active work behavior. Following the principle of reciprocity, individuals reciprocate beneficial behaviors when deriving satisfaction from another party. Paradoxical leadership prioritizes the interests of both the organization and the individual, prompting employees to respond with increased proactivity.

Second, superior-subordinate guanxi plays a mediating role between paradoxical leadership and employees’ proactive behaviors. In the Chinese context, SSG serves as a significant organizational resource and is typically the primary factor employees consider before engaging in behaviors (Qian et al., 2018). This favorable relationship is often established through shared values or similar interests and fosters closeness, promoting proactive behaviors regardless of the context’s formality. When employees’ active behavior is acknowledged, it can enhance their motivation, resulting in a positive cycle (Bakar and McCann, 2014). This ongoing process of communication, acceptance, and feedback gradually infuses flexibility and vitality into the organization. Additionally, employees’ sense of identification and dependence on the organization intensifies, inspiring them to actively and synergistically contribute to the organization while encouraging their peers to do the same.

Third, self-efficacy plays a mediating role between paradoxical leadership and employees’ active work behaviors. The premise for employees taking initiative in their work behavior is trust in the organization and confidence in their own abilities (Jung et al., 2022). Once individuals commit to proactive work behavior, they must feel capable of executing it successfully, known as self-efficacy. The organization must provide an appropriate platform to support the completion of these tasks (Peura et al., 2021). Paradoxical leadership values the strengths and weaknesses of each employee, treats all individuals equally, and shows respect for others. By promoting confidence and reducing overthinking, leaders can encourage active work behavior.

Fourth, self-efficacy plays a mediating role between superior-subordinate guanxi and employee’s active work behavior. High-quality supervisor-subordinate guanxi can promote a sense of security among employees within the organization. They no longer need to constantly worry about disapproval from supervisors or the judgment of peers. This can reduce anxiety, encourage forward-thinking, and promote active engagement in work (Freire et al., 2020). Furthermore, social cognitive theory suggests that strong supervisor-subordinate guanxi fosters greater trust in the organization among employees, leading to improved communication and ultimately a deeper understanding of tasks. This increased understanding promotes confidence in employees’ ability to complete tasks, encouraging proactive work behavior.

Fifth, superior-subordinate guanxi and self-efficacy exert a chain mediating effect between paradoxical leadership and employees’ proactive work behaviors. That is to say, paradoxical leadership—supervisor-subordinate guanxi–self-efficacy—employees’ proactive work behaviors. We found that the application of paradoxical leadership enhances superior-subordinate guanxi by treating subordinates as equals and facilitating communication with them. This approach provides employees with great satisfaction and a sense of self-efficacy (Han et al., 2023), leading to proactive work behavior.

### 5.2 Theoretical implications

First, additional adjustments were made to the precursor variables of employees’ provocative work behavior and the prognostic outcomes of paradoxical leadership. The literature on the correlation between paradoxical leadership and employees’ proactive work behavior is scarce, with experts mostly concentrating on employees’ provocative work behavior in a single role (Han et al., 2023), which gives a rather restricted viewpoint. By utilizing Parker and Collins' (2010) four-dimensional scale, which covers employee roles both within and beyond specific duties, it was confirmed that paradoxical leadership can significantly and positively predict employee proactive behaviors in these capacities. This enhances the predictive ability of the paradoxical leadership model and broadens the range of influential factors on employee proactive behaviors, thereby enriching the related theoretical research.

Second, this study reveals the influence of paradoxical leadership on employees’ active work behavior. Empirical analysis supports the model which shows that all dimensions of paradoxical leadership positively impact employees’ self-efficacy by enhancing subordinate-supervisor guanxi, thereby promoting proactive behaviors among employees. Moreover, the model confirms that paradoxical leadership has a significant effect on both SSG and self-efficacy (Wu and Ma, 2022). It demonstrates that harmonious supervisor-subordinate guanxi leads to an increase in proactive behaviors among employees (Liu et al., 2021). This study sheds light on the mechanism by which paradoxical leadership affects proactive work behavior among employees, revealing previously unknown information about this phenomenon (Liu et al., 2021). The research contributes to existing theoretical literature on proactive work behavior, paradoxical leadership, superior-subordinate guanxi, self-efficacy and social exchange in the fields of management and psychology.

### 5.3 Practical implications

Through empirical research, we have confirmed the impact of paradoxical leadership on employees’ proactive work behavior and the underlying transmission mechanism. This discovery has significant implications for promoting proactive behaviors among employees in their roles and beyond, and offers valuable guidance for business management practices. The following recommendations are primarily included:

First, we should place greater emphasis on building harmonious superior-subordinate guanxi. Evidenced by the empirical examination results, it is clear that supervisor-subordinate guanxi has a positive impact on employees’ provocative work behaviors. Establishing a strong supervisor-subordinate guanxi is crucial for enhancing employee proactivity. However, building such relationships can be a difficult task in management practice. Managers aim to satisfy employees’ needs for self-fulfillment, respect, and a sense of belonging, making them feel indispensable to the organization. It is important for managers to share resources with employees whenever possible, as this can help broaden their work perspectives and improve communication. Giving proper credit to employees for their achievements, while avoiding overshadowing them with personal competencies, can generate feelings of security and accomplishment, ultimately motivating and boosting their enthusiasm. Managers should follow conventional structure by including common academic sections and maintaining regular institution and author formatting. They should utilize clear, objective, and value-neutral language, avoiding biased or emotional wording and passive tone, with consistent technical terms and sentence structure. Managers should explain technical term abbreviations when first using them. They should keep language formal, avoiding contractions, colloquial words, informal expressions, and unnecessary jargon, while making positions clear through hedging. The use of precise subject-specific vocabulary should be used when it conveys the meaning more precisely than a similar non-technical term. Finally, ensure the text is free from grammatical, spelling, and punctuation errors. Moreover, managers should aim for objectivity by avoiding subjective evaluations unless marked as such. They should prioritize comprehensibility and logical structure by using clear, concise language in simple sentences that flow logically with causal connections. The relationship between employees and their roles serve as the foundation and prerequisite for effective communication. Observational evidence indicates that employees who maintain regular contact with their leaders, both during and beyond work hours, demonstrate higher levels of work enthusiasm and greater self-efficacy. Such connections enable employees to gain a more holistic understanding of their managers and, by extension, their organization’s objectives, ultimately resulting in work outputs that align with the organization’s goals. Upon recognition by the organization, employees exhibit increased enthusiasm toward their tasks, creating a positive feedback loop. Building a strong sense of organizational support can enhance employees’ identity and trust in their workplace, subsequently leading to proactive behaviors both within and beyond their roles. Previous studies have demonstrated that establishing a strong supervisor-subordinate guanxi has a significant positive impact on employee work performance, loyalty, and constructive behaviors, such as providing feedback. By integrating the research presented in this paper with related studies in other fields, it becomes evident that establishing healthy SSG within an organization is critical and plays a pivotal role in organizational development.

Second, leaders must improve their abilities to handle paradoxical issues. Extant research indicates that there are several paradoxes in managerial practices that remain unresolved, specifically related to organizational focus, management vs. innovation, and team vs. individual incentives. These issues are critical and significantly impact organizational development, necessitating a paradoxical approach to leadership, thinking, and situations to comprehensively address them. This highlights the positive effect of paradoxical leadership on employees’ proactive behaviors. Paradoxical leadership balances contradictions between individuals and the group, uniformity and individualization, centralization and autonomy, adherence and flexibility, and closeness and distance. This balance satisfies both organizational and individual needs. Hence, improving managers’ paradoxical leadership skills, such as maintaining viewpoints while embracing varied opinions and delegating within centralized frameworks, is crucial in activating motivation, initiative, and cohesion to meet both individual and organizational requirements for high-quality progress.

Third, leaders should prioritize organizational development and cultivate a harmonious environment to establish a “safe” and proactive workplace for employees. As a significant factor that affects employees’ psychological fluctuations, paradoxical leadership can effectively neutralize differentiation between organizational and individual demands, creating an organizational atmosphere that combines guidance, support, and openness. This climate allows employees to exercise a personalized approach to work and appropriate decision-making authority, which can substantially boost their SE. Therefore, leaders should strive to adjust their management style, continuously optimize the organizational environment, and strengthen organizational construction. They should plan for the individual and overall development of employees scientifically and rationally. They should also pay attention to constructing team culture and emphasize positive feedback, which will have a positive impact on employees’ psychological cognition.

Fourth, leaders should focus on enhancing employees’ self-efficacy. Research indicates that self-efficacy has a positive impact on employees’ proactive work behavior. Therefore, it is essential for leaders to prioritize the improvement of employees’ self-efficacy within the organization. By addressing employees’ excessive worries and concerns, leaders encourage the promotion of proactive behaviors, which benefit the organization’s development. Additionally, employees exhibit a higher degree of proactive behaviors outside the parameters of their designated role. If the employees’ provocative behaviors are perceived as overstepping, they may lead to greater resistance in completing tasks. This may adversely affect their promotion and performance evaluation. Furthermore, offending colleagues’ interests may result in a lack of support during task completion, thereby exacerbating difficulties. Ultimately, all these issues can be attributed to a lack of organizational support, resulting in decreased self-efficacy, which causes employees to hesitate in their work behavior. Therefore, managers should begin by assigning tasks, carefully considering employees’ abilities and allowing them to showcase their individual capabilities, while also offering positive feedback and support for completing tasks. Furthermore, providing guidance and support to alleviate any work-related concerns for employees can lead to improved self-esteem and increase their proactive behaviors. It is important to offer timely assistance in order to enhance their confidence.

### 5.4 Limitations and future research

This study, while refining and supplementing related theories, serves as a valuable reference for enterprise management practices concerning employee motivation. However, due to external constraints and limitations on the researcher’s time, energy, and capability, there are inevitable shortcomings and areas of deficiency in the research. These research limitations are addressed below, accompanied by a look forward to future studies.

First, future research can reduce the common variance. Due to the covid-19, this research was conducted online, and data was not collected in a paired manner. Although privacy of responses and answerability of the questionnaire were emphasized during the design and survey process, and the significance of the common variance was tested, ensuring the research data does not suffer from severe common variance. However, in future research about leadership style and employee behavior, conditions allow for the adoption of leadership and employee pairing to collect data, taking full account of the diversity of data sources.

Second, future research will expand mediating mechanisms and moderating effects. Even though this study investigated the mediating effect and chain mediating effect of supervisor- subordinate guanxi and self-efficacy according to the social exchange theory and social cognitive theory, the future research can further attempt to delve into intrinsic mechanism of paradoxical leadership on employees’ proactive work behavior from other theoretical perspectives.

## Data availability statement

The raw data supporting the conclusions of this article will be made available by the authors, without undue reservation.

## Ethics statement

Ethical review and approval was not required for the study on human participants in accordance with the local legislation and institutional requirements. Written informed consent from the patients/participants or patients/participants legal guardian/next of kin was not required to participate in this study in accordance with the national legislation and the institutional requirements.

## Author contributions

QQ: Conceptualization, Data curation, Formal analysis, Investigation, Methodology, Writing – original draft. WX: Data curation, Formal analysis, Investigation, Writing – review & editing. SQ: Data curation, Investigation, Writing – review & editing.
